# Diagnosis of Bleeding Disorders in Adolescents Hospitalized for Heavy Menstrual Bleeding

**DOI:** 10.1055/a-1892-1987

**Published:** 2022-09-26

**Authors:** Lauren E. Amos, Ashley K. Sherman, Shannon L. Carpenter

**Affiliations:** 1 Division of Hematology/Oncology/Bone Marrow Transplant, Children's Mercy Hospital, Kansas City, Missouri, United States; 2 Department of Pediatrics, The University of Missouri-Kansas City, Kansas City, Missouri, United States; 3 Department of Health Services and Outcomes Research, Children's Mercy Hospital, Kansas City, Missouri, United States

**Keywords:** pediatric hemostasis, von Willebrand disease, menorrhagia, hemophilia

## Abstract

Hundreds of adolescents are hospitalized in the United States yearly with anemia due to heavy menstrual bleeding (HMB). Limited data exist regarding how these patients are evaluated and how many are diagnosed with a bleeding disorder. The aim of this study was to determine the prevalence of bleeding disorders in adolescents hospitalized for HMB. Secondary aims included identification of risk factors for severe anemia leading to hospitalization and the hematology assessment. This was a retrospective cohort study of patients aged 9 to 21 years hospitalized for HMB and anemia at a tertiary care children's hospital from January 1, 2000, to December 31, 2017. A total of 118 girls hospitalized for HMB and anemia were included. Almost 30% of patients were African American. Hematology involvement did not occur in 42% of patients. Sixty patients completed bleeding disorder testing and 57% (34/60) were diagnosed with a bleeding disorder. Most patients diagnosed with von Willebrand disease (VWD) tested while hospitalized and anemic had VW levels <100%A but 9/25 (36%) girls not evaluated by Hematology also had VW levels <100%. Despite an established Inpatient Coagulation Consult service, many adolescents hospitalized with HMB did not receive an appropriate evaluation for bleeding disorders. African Americans were disproportionately hospitalized for HMB. VW levels are elevated with HMB and severe anemia, but levels <100 seem to be predictive of VWD in this setting. Further research is needed to determine optimal timing of bleeding disorder evaluation, but many adolescents hospitalized for HMB may have an underlying bleeding disorder.

## Introduction

Heavy menstrual bleeding (HMB) occurs in up to 40% of adolescent females. Many adolescents are hospitalized with HMB and severe anemia every year in the United States. 1 2 As hospitalization for HMB is largely preventable, it is important to identify those patients who are at risk for this occurrence. Limited data suggest that African American and Hispanic females are disproportionately admitted for HMB as compared with Caucasians. 2 It can be difficult to predict which adolescents admitted for HMB will ultimately have a bleeding disorder, and prevalence of bleeding disorders in these patients is not well known but may be as high as 30%. 3 Factors such as demographics, bleeding or clinical symptoms, or laboratory values have not been shown to differ between girls eventually diagnosed with a bleeding disorder versus those without a bleeding disorder. 4 Other variables such as younger age at presentation, Hispanic ethnicity, and more severe bleeding symptoms may increase the likelihood of a bleeding disorder in this population. 5 Provider determination of which patients to screen is also variable and is associated with factors such as proximity to a hemophilia treatment center, younger age, and commercial insurance. 6

The most common bleeding disorders to cause HMB are von Willebrand disease (VWD) and platelet function disorders (PFDs). 7 8 Guidelines from the American College of Obstetricians and Gynecologists (ACOG) and the National Heart, Lung, and Blood Institute (NHLBI) recommend evaluation for bleeding disorders in patients with HMB. 9 10 Testing should be done with guidance of a Hematologist and include laboratory screening for VWD, PFDs, factor deficiencies, and disorders of fibrinolysis. 9 Despite these guidelines, most adolescents admitted with HMB do not receive Hematology consults and hemostatic screening varies widely while inpatient. 2 11

Testing for VWD in hospitalized patients who are severely anemic and actively bleeding is also problematic. von Willebrand factor (VWF) is an acute phase reactant and increases in response to stress. Testing young women while hospitalized for anemia may not reflect their physiologic VWF levels and miss the diagnosis of VWD. 12 13 Many Hematologists may choose to defer von Willebrand testing during this time frame and repeat it in the outpatient setting once anemia resolves. Although recent data suggest that patients with von Willebrand antigen and/or activity >100% on initial testing are unlikely to have VWD, these studies did not specifically evaluate the effect of anemia on VWD testing. 12 14

Failure to test for PFDs may miss a large proportion of patients with these disorders. The modality of testing is also important, as some patients are tested with the PFA-100 alone. The PFA-100 is considered a screening tool for PFDs but is not diagnostic or specific, and can give false-negative results, leading to missed diagnosis of PFDs. In addition, it is affected by many variables including low hematocrit, which can produce inaccurate results. 15

More data are needed to identify which adolescents with HMB are at risk for hospitalization and which patients will have underlying bleeding disorders. In addition, standardized Hematology inpatient involvement and posthospitalization evaluation for bleeding disorders in these patients is necessary to avoid missing or delaying the diagnosis. This study sought to identify risk factors for HMB hospitalization, assess inpatient management, and determine prevalence of bleeding disorders in adolescents hospitalized for HMB. As most studies have focused on the inpatient variation in management, we also sought to determine how these adolescents were referred to Hematology and evaluated for bleeding disorders after hospital discharge. To our knowledge, this is the largest inpatient cohort of adolescents with HMB and outpatient follow-up studied to date.

## Materials and Methods

### Study Design and Subjects

A retrospective cohort study was performed at our institution, which is a free-standing tertiary academic center, from January 1, 2000, to December 31, 2017. Patients were identified by a registry kept by the Children's Mercy Hemophilia Treatment Center, by reviewing patients seen at the Young Women's Clinic, which is a comprehensive clinic staffed by pediatric Hematologists and Gynecologists for adolescent females with HMB and bleeding disorders, and by performing an electronic medical record (EMR) query of admission and discharge ICD-9 and ICD-10 diagnosis codes of HMB and anemia. **Appendix 1** lists all diagnosis codes used. Participants were aged 9 to 21 years and admitted to the hospital with a diagnosis of anemia (hemoglobin below the lower limit of normal for age) and HMB. HMB was defined as a period greater than 7 days and/or use of more than seven tampons or pads per day during menses and/or pictorial bleeding assessment score (PBAC) greater than 100. The PBAC is a validated tool to assess HMB in females. 16 Participants were excluded if hemoglobin was normal for age or if they did not meet inclusion criteria for HMB.

### Data Collection

Data collected included age at time of hospitalization, race, ethnicity, zip code, and presence of HMB since menarche. Zip codes were used as proxies for socioeconomic status by linking to available zip code level data from the 2010 U.S. census. 17 We used zip codes to link to median household income, percentage of population living below poverty rate, and population. Laboratory data obtained were hemoglobin at time of hospital admission, von Willebrand antigen and activity, PFA-100, and whole blood platelet aggregation with secretion testing. Repeat testing of von Willebrand antigen and activity after hospital discharge was also recorded. Treatment modalities included were receipt of packed red blood cell transfusion, oral iron therapy, oral tranexamic acid (TXA), and hormonal therapy, which consisted of intravenous estrogen, combined oral contraceptive pills, oral progesterone, or medroxyprogesterone acetate.

### Coagulation Testing and Diagnostic Methods

von Willebrand testing was performed at our institution and included von Willebrand antigen and plasma-derived factor VIII assay. Prior to 2018, ristocetin cofactor was used to assess von Willebrand activity. After 2018, von Willebrand activity assay using binding of monoclonal antibody to the VWF A1 epitope was used. PFA-100 was also run at our institution. Whole blood impedance aggregometry was performed at a local specialized coagulation laboratory to diagnose PFDs. Hematology consultation and outpatient clinic notes, and hematologic laboratories were reviewed to determine diagnosis of a bleeding disorder. Diagnosis of a PFD was confirmed with platelet aggregation with secretion testing on two separate occasions. Diagnosis of VWD was confirmed with two sets of VWD testing on separate occasions.

### Hematology Consultation

We determined whether Hematology was consulted by reviewing medical documentation including emergency department, admission, progress, consultation, and discharge notes. Presence of outpatient Hematology referral was determined by reviewing scheduled outpatient appointments, outpatient Hematology phone messages, and outpatient Hematology clinic notes.

### Institutional Review Board Approval and Statistical Analysis

This protocol was reviewed and approved by the Children's Mercy Institutional Review Board. Informed consent and HIPAA authorization were waived, as the study utilized deidentified retrospective data to perform the study. Statistical analysis was performed using SAS, version 9.4 (SAS Institute, Inc., Cary, NC). Means, medians, standard deviations (SDs), interquartile ranges (IQRs), and proportions were used to summarize the data. Chi-square tests were used to compare groups on categorical variables, and *t* -tests or Kruskal–Wallis tests were used to look for differences in continuous variables. Associations between categorical variables were analyzed using Pearson's correlations. A significance level of 0.05 was used for all analyses.

## Results

### Demographics

In total, 118 females were hospitalized for HMB and anemia over the study period. Table 1 displays a summary of patient characteristics. Mean patient age was 13.8 years (SD, 1.93; 95% confidence interval [CI], 10–19). Most patients were white (69%), followed by African American (29%) and Hispanic (8%). Median household income was 49,851.50 US dollars (IQR, 36,871–71,657), and median percentage below poverty rate was 13.6 (IQR, 6.4–21.8). 17

**Table 1:** Patient characteristics

Age (mean), y	13.8 (range, 10–19)	Number of patients (%)	Number of patients (%)
Race (n, %)	White	67 (56)	
	Black	34 (29)	
	Hispanic	10 (8)	
	Not listed	5 (4)	
Mean hemoglobin at admission, g/dL	6.2 (range, 2.4–11.9)		
Treatment modalities (n, %)	Packed red blood cell transfusion	96 (81)	
	Oral iron	95 (80)	
	Oral TXA alone	6 (5)	
	Hormonal therapy alone	76 (64)	
		Type of Hormonal Therapy ( *n* = 102)	
		Intravenous estrogen	33 (32)
		Combined oral contraceptives	48 (47)
		Oral progesterone	12 (12)
	Hormonal therapy + TXA	26 (22)	
Procedures (n,%)	Balloon tamponade	3 (3)	
	Intrauterine foley	1 (1)	
	Dilatation and curettage	3 (3)	

Abbreviation: TXA, tranexamic acid.

### Treatment

Mean hemoglobin at time of hospital admission was 6.2 g/dL (SD, 1.8; 95% CI, 2.4–11.9). Only 38/102 (37%) patients had laboratory assessment of iron deficiency while hospitalized. Of those, 35/38 patients had ferritin levels and all were severely iron deficient with ferritin less than 13 ng/mL. All patients who had iron studies obtained were started on oral iron. Hormonal therapy was used as single therapy in 76 patients and in combination with TXA in 26 patients. Type of hormonal therapy consisted of combined oral contraceptives (48/102; 47%), intravenous estrogen (33/102; 32%), and oral progesterone (12/102; 12%). TXA was used with combined oral contraceptives in 14 patients. All patients except one received TXA orally. Most patients required packed red blood cell transfusion and were treated with oral iron. No patients received intravenous iron while hospitalized. Seven patients required procedural therapy for cessation of menstrual bleeding. Table 1 lists demographic and treatment data.

### Referral to Hematology

Hematology referrals in the form of either an inpatient consultation or an outpatient clinic referral were present in 68/118 (58%) patients, and 60 of those patients were followed up as an outpatient. Patients who were younger were more likely to be referred to Hematology (mean age, 13.5 vs 14.4 years, *p* = 0.01). HMB since the onset of menarche was reported in 52 (44%) patients. Of those with HMB since the onset of menarche, 35/52 (51%) patients were referred to Hematology and 15 were diagnosed with a bleeding disorder. PFDs were diagnosed in 10 patients and VWD was diagnosed in 5 patients. Eleven patients with HMB since menarche had negative evaluations for bleeding disorders. The presence of HMB since menarche was not associated with increased likelihood of referral to Hematology ( *p* = 0.61). There was also no significant difference in severity of anemia at presentation and referral, as mean hemoglobin in referral group was 6.3 g/dL versus 6.2 g/dL in patients not referred ( *p* = 0.71). Although not statistically significant, 65% of white patients were referred to Hematology as opposed to 52% of African American patients ( *p* = 0.08).

### Diagnostic and Hematology Evaluation Outcomes

**Fig. 1:**
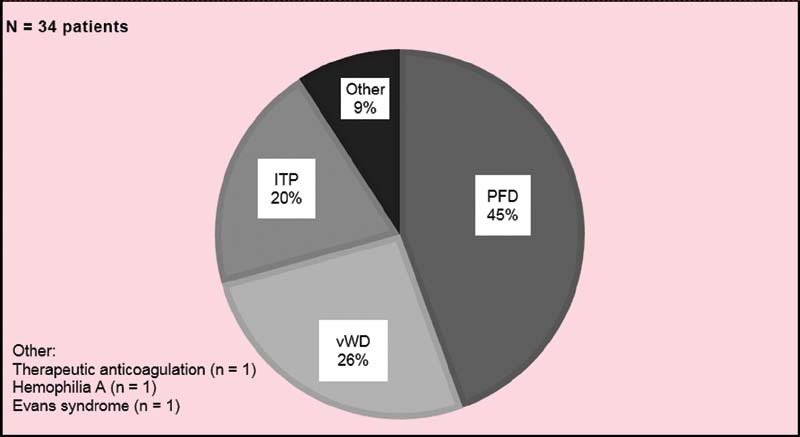
Bleeding disorder diagnoses in adolescents hospitalized for HMB.

A hematologic disorder was diagnosed in 34/60 (57%) females who completed a hematologic evaluation. The most common bleeding disorder diagnosed was a PFD, followed by VWD. All patients diagnosed with a PFD were diagnosed in the outpatient setting. To be diagnosed with a PFD, patients had two sets of whole blood impedance aggregometry that showed consistent defects. Adenosine diphosphate receptor defects were the most commonly diagnosed PFD and occurred in five patients. Three patients had platelet secretion defects, two patients had nonspecific defects, two patients had Bernard–Soulier syndrome, and one patient had a storage pool disorder. Patients met diagnostic criteria for VWD if von Willebrand activity or antigen levels were less than 50% given clinical bleeding symptoms. Of the patients diagnosed with VWD, 2/9 (22%) had von Willebrand antigen or activity levels >100 while hospitalized and anemic. No patients with VWD were identified by testing obtained during hospitalization ( Table 2 ). All patients were diagnosed with type 1 VWD. Most patients diagnosed with bleeding disorders were white. Only 3/15 patients diagnosed with a PFD were African American. The presence of HMB since menarche was not associated with diagnosis of a bleeding disorder ( *p* = 0.39). Severity of anemia as measured by initial hemoglobin less than 7 g/dL was also not indicative of having a bleeding disorder. Mean hemoglobin at hospital admission in girls diagnosed with a bleeding disorder was 6.28 g/dL as compared with 6.33 g/dL in girls who were not diagnosed with a bleeding disorder ( *p* = 0.92). Other hematologic disorders included immune thrombocytopenia, Evans syndrome, and thrombosis on therapeutic anticoagulation. For patients with immune thrombocytopenia, all patients except one had severe thrombocytopenia with platelet counts less than 5,000/μL. Fig. 1 depicts all hematologic disorders identified.

**Table 2:** Diagnostic VWD laboratory data for patients with HMB and diagnosed with VWD

Hgb (g/dL)	H F8 (%)	H VW Ag (%)	H VW Act (%)	O F8 (%)	O VW Ag (%)	O VW Act (%)
8.3	282	119	120	68	58	27
6.4	183	88	85	110	42	34
8.6	151	51	NO	66	34	29
10.2	62	39	36	49	30	20
4.5	252	100	67	81	48	42
7.3	282	185	92	142	83	45
11.8	72	54	61	71	46	49
7.5	104	NO	NO	104	20	27
7.7	NO	NO	NO	21	20	12

Abbreviations: Ag, antigen; H, labs obtained while hospitalized; Hgb, hemoglobin; NO, not obtained; O, labs obtained outpatient; VW, von Willebrand; F8, factor activity; act, activity.

### Adolescents Not Evaluated by Hematology

A total of 42% of girls were not referred to or seen by Hematology. Hematologic screening in this group was variable but all patients had prothrombin time and activated partial thromboplastin time obtained and results were within normal range. VWD screening was obtained in 29 patients while they were hospitalized and anemic, and 9/29 (31%) had von Willebrand antigen and/or activity levels less than 100%. Testing was repeated in four patients as outpatients. PFD screening occurred in 10 patients, but the majority (8/10) had PFA-100 testing only and only 2 of those patients had platelet aggregation with secretion testing.

### Risk Factors for Severe Anemia

We also evaluated whether certain variables such as age, socioeconomic status, or residence in a rural area were associated with more severe anemia, which would increase the need for hospitalization. Socioeconomic status was determined by zip code median income and percentage of population in that zip code below the poverty level. Rural area was determined by zip code population. Age and socioeconomic status were not correlated with lower hemoglobin at presentation ( *p* = 0.42 and 0.26, respectively). Residence in a more populated or more urban area was correlated with lower hemoglobin at initial presentation ( *p* = 0.01; *r* = –0.12). Ethnicity was associated with significant differences in percentage of population below the poverty level ( *p* < 0.001) and income level ( *p* < 0.001). The median percentage of population below the poverty levels was 21% in African American females as opposed to 8% in white females. Median income of zip codes for African American females was 41,296 dollars versus 59,058 dollars for white females. 17

## Discussion

Adolescents who were hospitalized at our institution for severe anemia and HMB were disproportionately African American as compared with our region's racial distribution. Twenty-nine percent of females were African American as compared with the racial distribution of the Kansas City region, Missouri, where 12% of the population is African American. 17 Mean household income and median percentage below poverty rate for these patients were similar to the mean household incomes and median poverty rates in Kansas and Missouri. 17 However, African American females who were hospitalized lived in zip codes with higher poverty rates and lower income levels as compared with white females. This racial disparity has been demonstrated previously where African Americans represented up to a third of patients hospitalized for HMB. 2 11 This elevated rate of African Americans was not due to an increased number of PFDs, which have been reported to be more common in this group, as only three patients diagnosed with PFDs were African American. 18 In contrast, only 17% of adolescent females who were referred to a Hematology clinic for outpatient evaluation of HMB were African American. 19 Given that African American females were more likely to live in areas with lower socioeconomic variables in our study, this racial disparity may be due to limited access to health care, resulting in delayed interventions to address HMB. This may also be why urban residents presented with a significantly lower hemoglobin at the time of admission. African American women experience higher rates of HMB and their symptoms may be discounted by medical providers. 20

Racial disparities are seen in other conditions for African American adolescents such as asthma and type 1 diabetes, where hospitalizations rates are disproportionately higher. Community-based interventions have been successfully trialed to decrease rates of emergency room visits and hospitalizations for African Americans adolescents with asthma. 21 More research is needed to fully understand racial disparities leading to HMB hospitalizations for African American girls so that interventions can be developed.

We have an established Inpatient Coagulation Consult Service at our institution dedicated to patients with disorders of hemostasis and thrombosis. Our institution also has Care Process Models developed in collaboration with Pediatric Gynecology for the evaluation of HMB in patients admitted to the hospital. Surprisingly, despite this infrastructure, our patients lacked standardized inpatient evaluation and outpatient follow-up for bleeding disorders. Almost half of the patients in our study were not evaluated by Hematology while hospitalized or in the outpatient setting. Factors such as severity of anemia upon admission or HMB since menarche were not associated with referral to Hematology. This is concerning as these patients should have been evaluated for bleeding disorders as per national guidelines from ACOG and the NHLBI. 9 10 When patients were evaluated by Hematology, over half were diagnosed with a bleeding disorder. This is higher than prior studies that have evaluated the prevalence of bleeding disorders in adolescents hospitalized for HMB and more consistent with prevalence of inherited bleeding disorders in patients referred to Hematology clinics for HMB ( Table 3 ). Our study highlights the importance of completing a hematologic evaluation for these patients to avoid delayed or missed diagnoses. Delay in diagnosis remains a significant issue for women with bleeding disorders, as the average age of diagnosis is around 35 years. 22 Further multi-institutional research is needed to understand why a significant number of patients with HMB are not evaluated for bleeding disorders despite institutional and national guidelines.

**Table 3:** Prevalence of bleeding disorders in patients with heavy menstrual bleeding in outpatient or inpatient outings

Authors	Patient location	No. of patients who completed testing for bleeding disorders	No. (%) of patients diagnosed with bleeding disorder
Zia et al, 2020	Outpatient	200	67 (33)
O'Brien et al, 2019	Outpatient	77	27 (35)
Zia et al, 2018 27	Outpatient	248	72 (29)
Alaqzam et al, 2018 28	Outpatient	73	34 (36)
Díaz et al, 2014	Outpatient	131	69 (53)
Seravalli et al, 2013 29	Outpatient	113	54 (48)
Vo et al, 2013	Outpatient	105	65 (62)
Bevan et al, 2001 30	Inpatient and outpatient	14	8 (57)
Rosen et al, 2020	Inpatient	44	12 (27)
Kanbur et al, 2004 31	Inpatient	47	3 (6)
Oral et al, 2002	Inpatient	25	8 (32)
Smith et al, 1998 32	Inpatient	37 (46 admissions)	11(24)
Claessens and Cowell, 1981 33	Inpatient	59	7 (12)

a Only evaluated for VWD, no other inherited coagulation disorders.

Given the challenges of identifying which adolescents with HMB will have an underlying bleeding disorder, use of a bleeding disorder assessment tool such as the International Society on Thrombosis and Haemostasis-BAT (bleeding assessment tool) (ISTH-BAT) may be a helpful tool. In adolescents with HMB, the ISTH-BAT was shown to be highly specific in diagnosing bleeding disorders when a higher cutoff score of greater than 4 was used. 23 Standardized implementation of a bleeding disorder assessment tool has not occurred at our institution but represents a promising way to further risk stratify patients with HMB.

Testing for VWD remains challenging due to fluctuations of VWF in response to stress. The effect of iron deficiency and severe anemia on von Willebrand testing is not well reported. Emerging data suggest that iron deficiency may obscure a diagnosis of VWD due to elevations in VWF. 24 Brown and colleagues recently published a retrospective cohort study evaluating VWF antigen levels during acute bleeding and then repeating testing with resolution of bleeding, which demonstrated that factor VIII and VWF are elevated due to HMB. In total, 70% of patients diagnosed with VWD met diagnostic criteria with their inpatient laboratories and VWF levels >100% had high negative predictive value. 25 Our study also demonstrated that most patients diagnosed with VWD had values <100% while inpatient and anemic. However, two patients eventually diagnosed with VWD had very elevated factor VIII levels and vW antigen or activity levels >100% while inpatient ( Table 3 ). In addition, 30% of patients not evaluated by Hematology had inpatient VWD testing with levels <100%. These patients did not have repeat testing once HMB resolved, and hemoglobin normalized. If testing had been repeated, they may have met diagnostic criteria for VWD. More evidence is needed to truly determine which patients need retesting to not miss adolescents who are hospitalized with HMB and have VWD despite elevated VWF levels while anemic.

Most patients received packed red blood cell transfusions as compared with other studies where the percentage of girls with severe anemia receiving transfusions for HMB ranged from 43 to 73%. Iron therapy was given more consistently, as 80% of patients received iron therapy or were prescribed iron at discharge. 2 11 26 Twenty percent of patients who were likely iron deficient did not receive iron therapy. This may be due to the large number of patients who were not assessed for iron deficiency while hospitalized. Evaluation of iron deficiency and treatment also needs to be standardized for this population. Hormonal therapy was used frequently but the type of hormonal therapy was variable. TXA was used in less than a third of our patients. Although TXA has only been approved to treat HMB in women since 2009, a large inpatient database study from 2012 to 2015 showed similarly low rates of use in this population at 8%. 2 27

This variability is not surprising as evidence is lacking on how to best treat HMB in adolescent females with and without bleeding disorders and standard of care has not been established. 22 Variations in treatment of HMB in hospitalized adolescents may result in longer hospital stays due to uncontrolled bleeding. 11 The efficacy of HMB management for hospitalized adolescents needs to be further studied so that consensus guidelines can be created. This is important to optimize care for these patients and decrease treatment failure and inpatient hospital stays.

Limitations of our study include the relatively small number of patients included from a single institution, which may reflect why certain variables such as HMB since menarche or severity of anemia at presentation were not predictive of a bleeding disorder. We also did not collect data regarding diagnosis of gynecological disorders, which may have contributed to why certain patients did not have hematologic evaluations for their HMB. The study is also limited by its retrospective nature. Although thorough chart review likely captured most of the referrals to Hematology, patients may have discussed via phone with Hematology without an official consult. It would still be expected that those patients would be seen by Hematology as an outpatient for further evaluation so referral to Hematology would still be captured, though it is possible some patients may have been lost to follow-up. This was a single-institution study, so it is possible that increased diagnosis of PFDs is due to institutional testing practices. This study would be strengthened by future prospective multicenter observational studies in adolescents hospitalized for HMB.

## Conclusions

Adolescents are at risk for severe anemia due to uncontrolled HMB. African American teenagers are admitted at higher rates compared with racial distribution for our geographical area. Further research is needed to understand why African American adolescents are hospitalized more frequently than other races for HMB and to identify additional risk factors for hospitalization for HMB. Increased awareness of bleeding disorders in females, institutional guidelines, and national recommendation for bleeding disorder evaluation in these patients did not ensure that appropriate testing for bleeding disorders was completed. When coagulation testing is completed for these patients, many have inherited bleeding disorders. Testing for VWD while experiencing HMB and severe anemia may result in elevated VWF levels that delay or miss the diagnosis. Optimal timing for VWD testing in this population remains to be determined but testing once bleeding and anemia resolves ensures that the diagnosis of VWD is not missed.

## Notes

Appendix 1
**ICD-9 Diagnosis Codes**

626.2 Excessive Menstruation
626.3 Pubertal Menorrhagia
626.8 Menstrual Disorder NEC
280.0 CHR Blood Loss Anemia
280.9 Iron Deficiency Anemia NOS
285.1 AC Posthemorrhagic Anemia
285.9 Anemia NOS

**ICD-10 Codes**

N92.0 Excessive and frequent menstruation with regular cycle
N9.3.8 Other specified abnormal uterine and vaginal bleeding
D50.0 Iron deficiency anemia secondary to blood loss (chronic)
D50.8 Other iron deficiency anemias
D50.9 Iron deficiency anemia, unspecified
D62 Acute posthemorrhagic anemia
D64.9 Anemia, unspecified
